# Chlorpyrifos modulates the mouse gut microbiota and metabolic activity

**DOI:** 10.1016/j.envint.2024.109022

**Published:** 2024-09-19

**Authors:** Robert G. Nichols, Bipin Rimal, Fuhua Hao, Jeffrey M. Peters, Emily R. Davenport, Andrew D. Patterson

**Affiliations:** a Department of Biology, The Pennsylvania State University, University Park, PA, USA; b One Health Microbiome Center, Huck Life Sciences Institute, University Park, PA 16802, USA; c Department of Veterinary and Biomedical Sciences, Center for Molecular Toxicology and Carcinogenesis, The Pennsylvania State University, University Park, PA, USA

**Keywords:** Chlorpyrifos, Gut microbiome, Flow cytometry, EPA acute reference doses

## Abstract

The organophosphate chlorpyrifos is a commonly used pesticide for fruits and vegetables despite its association with neurotoxicity in humans. While some studies suggest that organophosphates may impact the gut microbiota, no studies to date have investigated the direct effect of chlorpyrifos on the gut microbiota with doses that approximate environmentally relevant dietary concentrations (EPA chronic reference dose: 0.3 μg/kg/day in humans and EPA acute reference dose: 5 μg/kg/day in humans). Thus, we examined the influence of chlorpyrifos on the gut microbiota by assessment of bacterial physiology and metabolism using flow cytometry, ^1^H NMR-based metabolomics, and changes in the cecal microbiota community with 16S rRNA amplicon sequencing and analysis. Chlorpyrifos did not directly damage bacteria but rather perturbed bacterial metabolism. Chlorpyrifos exposure to bacteria increased the concentration of amino acids, carbohydrates, and nucleic acids. The relative abundances of *Lactobacillus, Allobaculum, Roseburia*, and *Butyricicoccus* increased after exposure to chlorpyrifos. Analyses of the 16S rRNA gene amplicon data predicted decreased amino acid biosynthesis and nucleic acid degradation and increased glycolysis which was supported by ^1^H NMR-based metabolomics. Collectively, these results demonstrate that environmentally relevant doses of chlorpyrifos can impact the metabolic activity of isolated gut microbes which may result in an imbalance in overall gut metabolic activity.

## 1. Introduction

Chlorpyrifos is a common polychlorinated, organophosphate pesticide used to prevent crop damage in many fruits (e.g., apples, peaches), vegetables (e.g., bell peppers, cucumbers), and nuts (e.g., almonds) (Kynetec, 2019). Chlorpyrifos is also used to treat golf course turf, eliminate termites, and treat wood and wood products to prevent damage from insects (Reaves, 2020). According to the recent registration of the Environmental Protection Agency (EPA) review of chlorpyrifos (Docket Number EPA-HQ-OPP-2008–0850), the agricultural sector used over 5 million pounds of chlorpyrifos annually between 2014 and 2018 (Reaves, 2020). The major concern for chlorpyrifos exposure is due to its presence in drinking water caused by storm runoff from farms (Reaves, 2020). When used on plants and crops, chlorpyrifos evaporates within 10–14 days and because it aerosolizes, it can be found in dust and elsewhere within homes and the environment (Kamrin, 1997; Roinestad et al., 1993). Chlorpyrifos exposure is reported to cause neurological and neurodevelopmental defects in children and neonates, respectively (Rauh et al., 2006; Saunders et al., 2012). Due to the extensive annual usage of chlorpyrifos in food products in the United States and elsewhere, the effects of dietary chlorpyrifos continue to require assessment and evaluation.

Individuals are exposed to chlorpyrifos from different sources including their diet, such as from treated fruits and vegetables. For example, on average, a stalk of celery could contain 0.05–0.11 PPM of residual chlorpyrifos, while a tomato could have between 0.002–0.012 PPM of residual chlorpyrifos (USDA Pesticide Detection Program or PDP 2022 annual summary) (USDA, 2022), and chlorpyrifos has also been detected in human breast milk (Rovira et al., 2022). Exposure to chlorpyrifos can also occur due to its use in non-agricultural applications including golf course turf treatments, wood production, utility poles, and railroads (Reaves, 2020).

In the United States, chlorpyrifos was banned from residential use in 2001 (Reaves, 2020). Thus, chlorpyrifos exposure levels in humans are currently lower compared with those previously recorded (Reaves, 2020). In December 2022 the EPA revoked the use of chlorpyrifos in the agricultural sector but in November 2023 the Eighth Circuit Court of Appeals vacated the EPA decision (Red River Valley Sugarbeet Growers, 2023). The EPA has reported a reference dose (RfD) for chronic exposure of chlorpyrifos in adult males as 0.3 μg/kg/day and an acute RfD of 5 μg/kg/day as of 2009 (Chlorpyrifos Technical Fact Sheet). To date, few studies have examined chlorpyrifos toxicity (chronic or acute) at environmentally relevant concentrations. One such study investigated the effects of chlorpyrifos doses close to the RfD (doses ranged from 0.010 mg/kg to 0.16 mg/kg in rats) and discovered that low-dose exposure caused an increase in stress in mice due to DNA instability (Kopjar et al., 2018). Though this study used low chlorpyrifos doses, this group mainly focused on neurotoxicity and did not investigate other avenues of chlorpyrifos toxicity like the gut microbiome. A second study investigated the effects of chlorpyrifos on the digestive system using the SHIME system (**S**imulator of the **H**uman **I**ntestinal **M**icrobial **E**cosystem) at a dose of 1 mg of chlorpyrifos/day (Joly et al., 2013). This study noted changes in the overall community composition of the SHIME microbiome even at these low doses (Joly et al., 2013).

The gut microbiome has become an increasingly important target of investigation because it has been reported that gut microbes are critical for nutrient digestion (Tremaroli and Bäckhed, 2012), gut barrier function (Takiishi et al., 2017), immune system development (Zheng et al., 2020), and drug and xenobiotic metabolism (Swanson, 2015). Given the reversal of the ban of chlorpyrifos, more research is needed to determine the potential impact of this pesticide not only on host tissues but on the microbiome. At relatively high doses (2.5–10 mg/kg (Sun et al., 2023) and 5 mg/kg (Li et al., 2022; Liang et al., 2019)) it was reported that chlorpyrifos causes increased intestinal permeability and modulation of the gut microbiome community (Li et al., 2022; Liang et al., 2019; Sun et al., 2023) potentially leading to increased insulin resistance and obesity in mice (Liang et al., 2019). Consistent with this, mice exposed to soil microbes (rather than sterile mouse bedding) had a lower risk of developing obesity or insulin resistance after chlorpyrifos exposure than controls (Li et al., 2022). These studies illustrate the importance of microbes in modulating the host phenotype after high levels of chlorpyrifos exposure. These studies suggest that chlorpyrifos impacts bacteria at both the community structure and function levels. However, it is unknown whether these effects also occur at the lower, more physiologically relevant doses.

To further investigate how acute exposure of chlorpyrifos impacts the gut microbiota, we used flow cytometry, ^1^H NMR-based metabolomics, and 16S rRNA gene amplicon sequencing to examine changes in bacterial physiology and function in a well-controlled *in vitro* system. We found that chlorpyrifos does not directly damage bacteria but significantly perturbs bacterial metabolism as identified by quantitative ^1^H NMR approaches. Several bacterial genera were altered due to exposure to chlorpyrifos and predicted decreases in bacterial amino acid production and predicted increases in glycolysis were also noted compared to controls. These studies provide new mechanistic insight into how environmental chemicals impact gut bacteria.

## 2. Methods

### 2.1. Chemical reagents

Chlorpyrifos (PESTANAL, #45395, Millipore Sigma, St. Louis, MO) was dissolved in dimethyl sulfoxide (DMSO) to prepare a 1 mg/μl, 0.1 mg/μl and a 0.01 mg/μl stock solution and stored at 4°C until use. Dehydrated brain heart infusion broth (BD Biosciences, San Jose, CA) was prepared with L-cysteine (Sigma Aldrich, St. Louis, MO) and reconstituted with sterile water.

### 2.2. Mice

Ten 8-week-old male C57BL/6J mice (Jackson Laboratory, Bar Harbor, Maine) were acclimated for one week prior to the start of the study. All animal studies were approved by the Institutional Animal Care and Use Committee at the Pennsylvania State University and were performed according to the university guidelines for the ethical treatment of animals.

### 2.3. Gut microbiota Isolation and incubation conditions

**Fig. 1.**
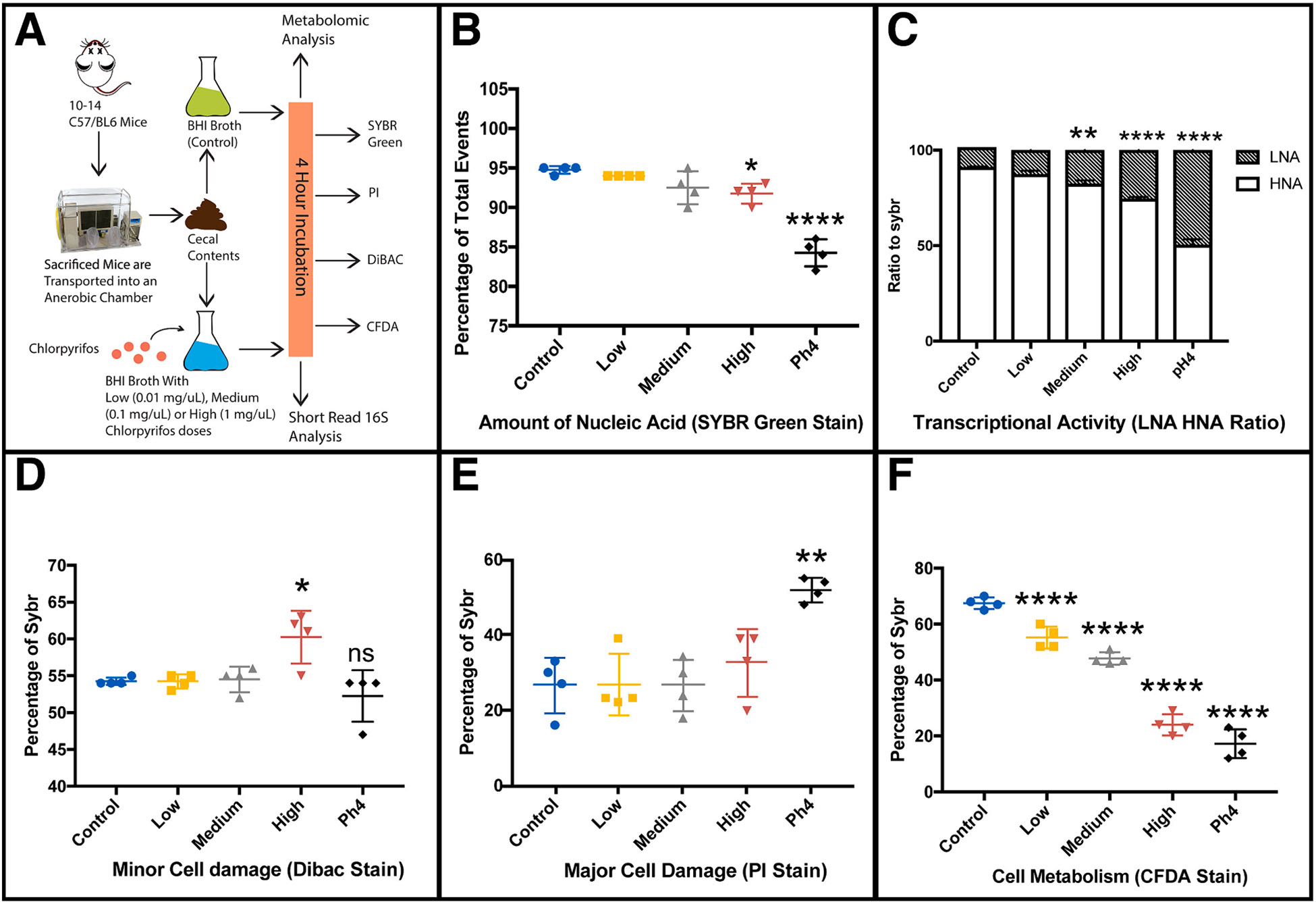
Flow cytometry demonstrates that chlorpyrifos affects the gut microbes through alterations in cell metabolism. **A)** Flow schematic showing a brief description of the setup of the flow cytometry experiment. **B)** Higher doses of chlorpyrifos result in reduced nucleic acid levels, seen by a decrease in the SYBR green stain. **C)** Medium and high chlorpyrifos doses result in decreased cellular replication and metabolism as seen by the shift in High Nucleic Acid (HNA) to Low Nucleic Acid (LNA) (obtained from the overall SYBR stain results). **D)** High chlorpyrifos doses result in an increase of minor cell damage (switch in membrane polarity) as seen by the increase in the DiBAC stain. **E)** No chlorpyrifos doses result in major cell damage (cell wall instability) as seen by the results of the PI stain. **F)** Chlorpyrifos inhibits cell metabolism in a dose-dependent manor, as seen by the results of the CFDA stain. All flow cytometry results shown are represented as percent of SYBR events (except for the SYBR stain which is percent of total events). Significance was obtained with 1-way ANOVA and Tukey’s Correction. *, p < 0.05; **, p < 0.01; ***,0.01; ***,p < 0.001; ****, P,0.0001. For all groups N=4 with PH4 group (12 M HCL treated group) acting as a positive control.

The ceca from ten, 8-week-old male C57BL/6J mice (Jackson Laboratory, Bar Harbor, Maine) were transferred to an anaerobic chamber (Coy Laboratory Products, Inc., Grass Lake, MI) and the contents were isolated and pooled. All of the following procedures were performed under anaerobic conditions with an oxygen level below 20 ppm. The microbiota incubation and flow cytometry preparation procedures were derived and modified from previously studies (Maurice and Turnbaugh, 2013; Cai et al., 2018; Tian et al., 2020) (see scheme in Fig. 1A). Briefly, brain heart infusion broth was added to conical tubes and a stock solution of chlorpyrifos was added to achieve three final concentrations of 0 (vehicle control, DMSO), 0.01 mg/mL (low), 0.1 mg/mL (medium) or 1 mg/mL (high). One tube contained brain heart infusion broth with 12 M HCl (pH4) as a positive control. Each suspension was prepared in duplicate (one for the flow experiment, the other replicate for ^1^H NMR-based metabolomics analysis as described below). Cecal contents were diluted 1:10 by adding 1.2 g of cecal contents to 12 mL of either the control tubes or tubes containing either low chlorpyrifos, medium chlorpyrifos, or high chlorpyrifos. The cecal content suspensions were incubated at 37 °C for 4 h in the dark and gently mixed at 300 rpm. After incubation, one set of samples was stored at −80 °C for ^1^H NMR analysis. The remaining samples were centrifuged (700xg, for 1 min) to remove debris and then 600 μL of the microbial supernatant were transferred to a new tube and then centrifuged (6000xg, for 3 min) to pellet the bacteria. The supernatant was discarded, and the pellet was washed with L-cysteine reduced PBS (resazurin was used as an oxygen indicator), centrifuged (6000 x g, for 2 min), and resuspended in reduced PBS. The wash step was repeated twice until the suspension was colorless and then diluted 120-fold with reduced PBS. The diluted suspension was transferred to a 1.5 mL tube for subsequent flow cytometry. The remaining suspensions were used for 16S rRNA amplicon sequencing analysis.

### 2.4. Microbial physiology profiling with flow cytometry

Flow cytometry was performed as previously described (Maurice and Turnbaugh, 2013; Cai et al., 2018). Briefly, four dyes were used to assess changes in bacterial physiology and damage. SYBR green was used to assess nucleic acid availability and replication (Gasol et al., 1999; Lebaron et al., 2001). Propidium iodide exclusion dye (Terstappen et al., 1988) and Bis-(1,3-Dibutylbarbituric Acid) Trimethine Oxonol (DiBAC) (Jepras et al., 1995) were used to assess major and minor cell damage, respectively. Carboxyfluorescein diacetate (CFSA) and carboxyfluorescein diacetate succinimidyl ester (CFSE) staining was used to assess bacterial metabolism (Hoefel et al., 2003). All flow cytometric analyses were performed with FlowJo software (V10, FlowJo, LLC).

### 2.5. ^1^H NMR metabolomics profiling

Frozen aliquots from the *in vitro* experiment were thawed and 1.0 mm diameter zirconia/silica beads were then added. Samples were homogenized at 6500 RPM for 60 s using the Precellys tissue homogenizer (Bertin Technologies, Rockville, MD). The homogenized samples were snap-frozen with liquid nitrogen and then thawed on a heat block at 37 °C. This freeze-thaw cycle was repeated two more times and then the samples were centrifuged to obtain the supernatant which was placed in a 2 mL Eppendorf tube. 1 mL of chilled MEOH:H20 (2:1) was added to the pellet. The samples were centrifuged at 3200 x g for 10 min. The ~ 2 mL supernatant was removed and added to the appropriate 2 mL tubes. The supernatants were dried down and then resuspended in 80 μL of PBS (K_2_HPO_4_/NaH_2_PO_4_, 0.1 M, pH 7.4, containing 100 % D2O and 0.29 M TSP-d4 as internal standard). Samples were centrifuged at 14000 x g for 10 min, and supernatants were transferred into 1.7 mm NMR tubes for NMR analysis.

^1^H NMR analysis was done as previously described (Cai et al., 2018; Tian et al., 2020; Tian et al., 2012). Briefly, 1D-^1^H spectra were acquired for all samples at 298 K using a Bruker Advance NEO 600 MHz NMR spectrometer (Bruker Biospin, Germany) operating at 600.15 MHz for proton. The standard noesygppr1d pulse sequence was used for recording ^1^H NMR experiments with presaturation water suppression during relaxation and mixing time. Following the simple preprocessing of phase, baseline in Topspin (Version 4.09, Bruker), all ^1^H spectra were imported into Chenomx NMR Suite (version 8.4, Chenomx Inc, Edmonton, AB, Canada) for metabolites profiling. Manual checking and adjustment were made for phase, baseline, and internal standard after automatic processing to ensure quality assurance. The individual metabolites were identified and quantified using the built-in metabolite library and the fitting algorithm in the Chenomx software, with concentrations determined relative to the internal standard (TSP, 0.29 mM). For performing PCA analysis, the 2075 bin values were obtained with equal binning (0.004 ppm) from 0.4 to 9 for each spectrum. Each binning was normalized to the internal standard (TSP). SIMCA 13.0 was used to create PCA plots (Umetrics, Sweden) based on the bin values above.

### 2.6. 16S rRNA gene amplicon sequencing analysis

Bacterial preparation and 16S rRNA gene amplicon analysis were performed as previously described (Nichols et al., 2018). Briefly, the reserved cell suspension from the flow cytometry experiment was centrifuged at 13,000xg for 5 min to pellet bacteria. Bacterial DNA was extracted using the OMEGA E.Z.N.A Stool Isolation kit. Isolated bacterial DNA was then normalized to 10 ng/μL for 16S V4 amplification (PCR conditions previously described (Nichols et al., 2018)). 16S rRNA gene amplification was assessed with a 1X agarose gel and samples were sent to the Pennsylvania State University Genomics core for Illumina MiSeq Nano paired-end sequencing resulting in ~ 600,000 raw reads. Sequencing results were returned and analyzed using mothur (v.1.47.0) resulting in bacterial relative abundance (Nichols et al., 2018; Kozich et al., 2013). ALDEx2 (v.1.32.0) was used to determine the significance of the differential bacterial relative abundance results (Fernandes et al., 2013).

### 2.7. PICRUSt2 analysis

Phylogenetic Investigation of Communities by Reconstruction of Unobserved States (PICRUSt2) (v.2.4.2) was used to predict bacterial functional changes (Douglas et al., 2020). An ASV table was created using the DADA2 (v.1.8) program in R (Callahan et al., 2016) and formatted for PICRUSt2 analysis by removing ASV sequences and converting the ASV count table into a biom file. The PICRUSt2 terminal command was then run on the obtained sequence file and the newly created biom file. These results were then collapsed into metacyc (Karp et al., 2018) superpathways and both the superpathway results and the individual pathway results were analyzed with DESeq2 (v 1.40.1) (Anders et al., 2010) to obtain statistically significantly different predicted pathways.

## 3. Results

### 3.1. Flow cytometry shows the direct effects of chlorpyrifos on the gut microbiota

To examine whether chlorpyrifos directly affects either the cellular integrity or metabolic activity of the microbiota, we performed flow cytometry on cecal contents incubated with varying concentrations of chlorpyrifos (0.01 mg/mL (low), 0.1 mg/mL (medium) or 1 mg/mL (high), with the pH4 group used as a positive control, see methods). First, we evaluated the effects of different dosages of chlorpyrifos on total nucleic acid and DNA replication in the microbiota, as measured by the uptake of SYBR green. Low and medium concentrations of chlorpyrifos had no effect on SYBR green uptake compared to controls (Fig. 1B). High-dose chlorpyrifos (1-way ANOVA with Tukey post hoc test, p = 0.02) and the acidic positive control (1-way ANOVA with Tukey post hoc test, p < 0.0001) caused a significant decrease in SYBR green uptake compared to the control group (Fig. 1B). We also investigated the ratio between high and low nucleic acid content, a decrease of this ratio indicates decreased levels of cellular division and metabolic activity (Zubkov et al., 2001; Bouvier et al., 2007). A dose-dependent decrease in high nucleic acid content and a significant increase of low nucleic acid content was observed in the medium (1-way ANOVA with Tukey post hoc test, p = 0.0038) and high (1-way ANOVA with Tukey post hoc test, p < 0.0001) dose chlorpyrifos groups compared to controls (Fig. 1C). The changes induced by medium and high dose chlorpyrifos in nucleic acids were also noted in the acidic positive control (1-way ANOVA with Tukey post hoc test, p < 0.0001) (Fig. 1C). Next, we evaluated whether chlorpyrifos damages bacterial cellular integrity. High-dose chlorpyrifos (1-way ANOVA with Tukey post hoc test, p = 0.01) exposure caused minor cell damage (change in membrane polarity) as assessed by increased DiBAC staining as compared to controls (Fig. 1D). There was a significant increase in the propidium iodide-stained cells only in the acidic positive control group (1-way ANOVA with Tukey post hoc test, p = 0.0006; Fig. 1E). By contrast, there was no significant change in propidium iodide-stained cells caused by exposure to chlorpyrifos indicating no significant major damage to the bacterial cell wall (Fig. 1E). Finally, we examined overall cellular metabolism by assessing CFDA uptake. A dose-dependent decrease of CFDA uptake was observed in all three chlorpyrifos treatment groups (low; 1-way ANOVA with Tukey post hoc test, p = 0.0008, medium; 1-way ANOVA with Tukey post hoc test, p < 0.0001, and high; 1-way ANOVA with Tukey post hoc test, p < 0.0001, Fig. 1F). A significant decrease of CFDA stain was also observed in the acidic positive control (1-way ANOVA with Tukey post hoc test, p < 0.0001, Fig. 1F). All samples were run randomly to account for CFDA degradation. For DiBAC, PI and CFDA stains, results are represented as percent of events stained for each dye out of the total amount of SYBR stained events for each treatment.

Taken together, these four dyes indicate that at high doses of chlorpyrifos, there are major perturbations in the gut microbiota, including decreased in total nucleic acids, decreased cellular replication, slight increases in minor cell damage, and decreases in cellular metabolism. However, at low and medium doses, only slight decreases of cellular replication and cellular metabolism were observed. Notably, major damage to bacterial cell walls was not detected at any chlorpyrifos concentration examined, demonstrating that even at lower concentrations, chlorpyrifos affects the gut microbiota primarily through a decrease in cellular metabolism rather than direct damage and compromised cellular integrity.

### 3.2. ^1^H NMR reveals bacterial metabolic changes after exposure to chlorpyrifos

**Fig. 2.**
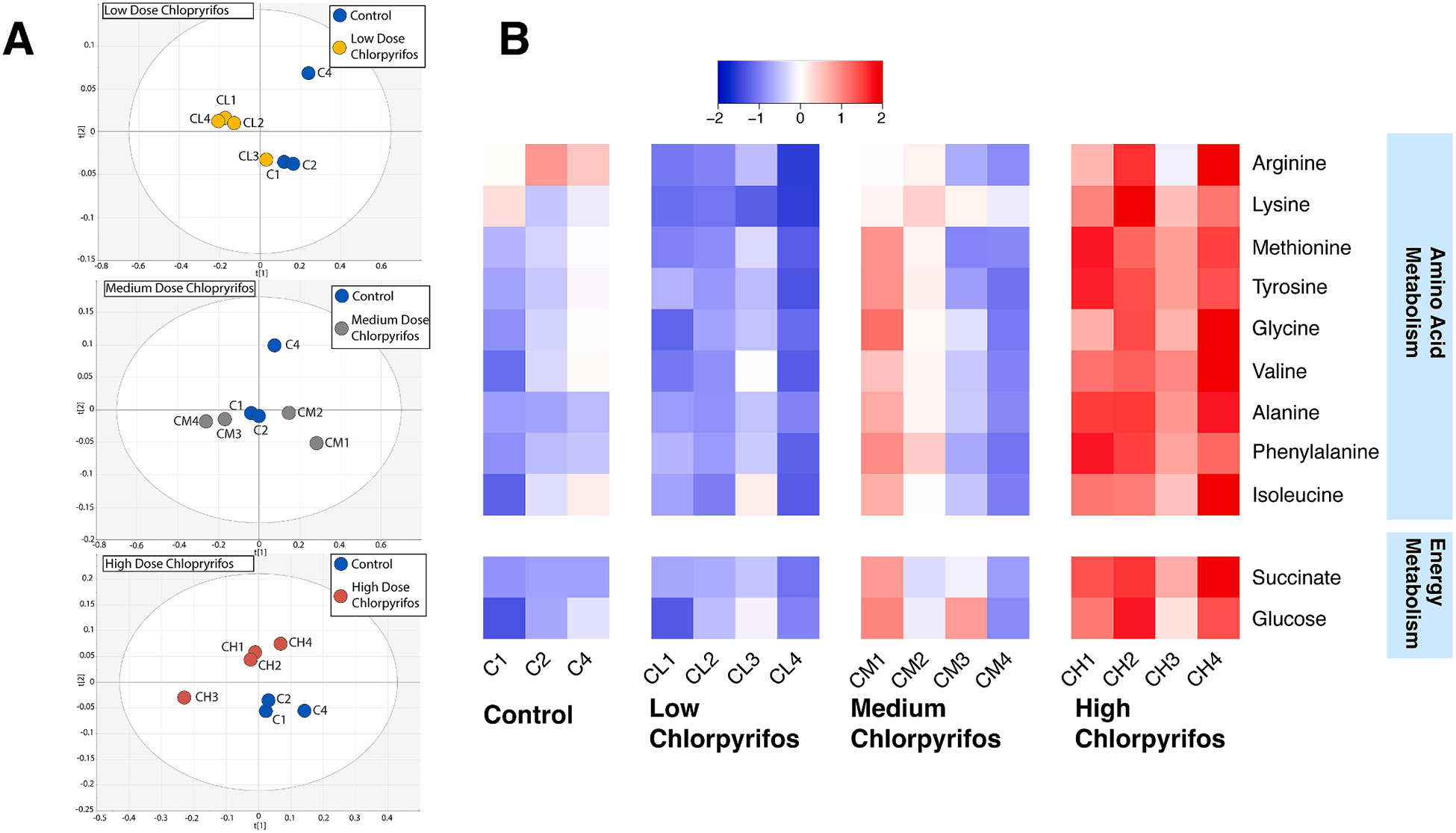
^1^H NMR demonstrates that chlorpyrifos alters the metabolic profile of gut microbes. **A)** Metabolomic shifts can be seen in the low-dose and high-dose chlorpyrifos groups as seen with the PCA score plots. PCA score plots were obtained from ^1^H NMR of metabolite profiling for the isolated cecal bacteria after exposure to the three doses of chlorpyrifos or vehicle. **B)** High-dose chlorpyrifos results in major increases of amino acids and glucose as seen in a heatmap of all significantly different metabolites. These metabolites are from isolated cecal bacteria after exposure to the three doses of chlorpyrifos or vehicle. Values are represented as Z scores with the equation Z=(x−μ)∕σ. Where x is the observed value, μ is the mean of the sample, and σ is the standard deviation of the sample. Red colors represent an increase compared to the average metabolite level and blue colors represent a decrease compared to the average metabolite level. All values are normalized to the internal standard TSP. Significance was determined to be P<0.05 after 1-way ANOVA with Tukey’s correction. See Supplemental Fig. 1 for exact metabolite graphs and individual significance.

Given the decrease in overall microbial metabolism observed at all concentrations of chlorpyrifos as measured by CFDA staining in flow cytometry, we next evaluated global metabolic differences between treatments and controls using ^1^H NMR. Principal component analysis (PCA) score plots show distinct clustering between control groups and low (0.01 mg/mL) and high-dose (1 mg/mL (high) chlorpyrifos (Fig 2A). Values used for these PCA plots are ^1^H NMR values normalized to the internal standard TSP. Low and medium-dose exposure to chlorpyrifos had little effect on metabolite concentration compared to control with one exception (Fig 2B). Significant metabolite changes were seen in arginine (1-way ANOVA, FDR corrected p value = 0.01), lysine (1-way ANOVA, FDR corrected p value = 0.0006), methionine (1-way ANOVA, FDR corrected p value = 0.004), tyrosine (1-way ANOVA, FDR corrected p value = 0.004), glycine (1-way ANOVA, FDR corrected p value = 0.01), valine (1-way ANOVA, FDR corrected p value = 0.004), alanine (1-way ANOVA, FDR corrected p value = 0.0006), phenylalanine (1-way ANOVA, FDR corrected p value = 0.005), lactate (1-way ANOVA, FDR corrected p value = 0.015), isoleucine (1-way ANOVA, FDR corrected p value = 0.015), succinate (1-way ANOVA, FDR corrected p value = 0.0006) and glucose (1-way ANOVA, FDR corrected p value = 0.015).

Tukey post hoc tests were done on the significant metabolites to assess which groups had significant increases or decreases when compared to the control group. Low-dose chlorpyrifos exposure resulted in a significant decrease in both arginine (Tukey post hoc test, p = 0.048) and lysine (Tukey post hoc test, p = 0.018) compared to controls (Fig. 2B). High-dose chlorpyrifos exposure resulted in a significant increase in glycine (Tukey post hoc test, p = 0.034), alanine (Tukey post hoc test, p = 0.0003), lysine (Tukey post hoc test, p = 0.007), methionine (Tukey post hoc test, p = 0.01), tyrosine (Tukey post hoc test, p = 0.009), phenylalanine (Tukey post hoc test, p = 0.005), valine (Tukey post hoc test, p = 0.006), and isoleucine (Tukey post hoc test, p = 0.037) compared to control (individual metabolite levels see Supplemental Fig. 1). High-dose chlorpyrifos also resulted in a significant increase in glucose (Tukey post hoc test, p = 0.0167) and succinate (Tukey post hoc test, p = 0.0003) (Fig. 2B).

### 3.3. 16S rRNA amplicon sequencing illustrates bacterial taxonomic shifts due to chlorpyrifos exposure

**Fig. 3.**
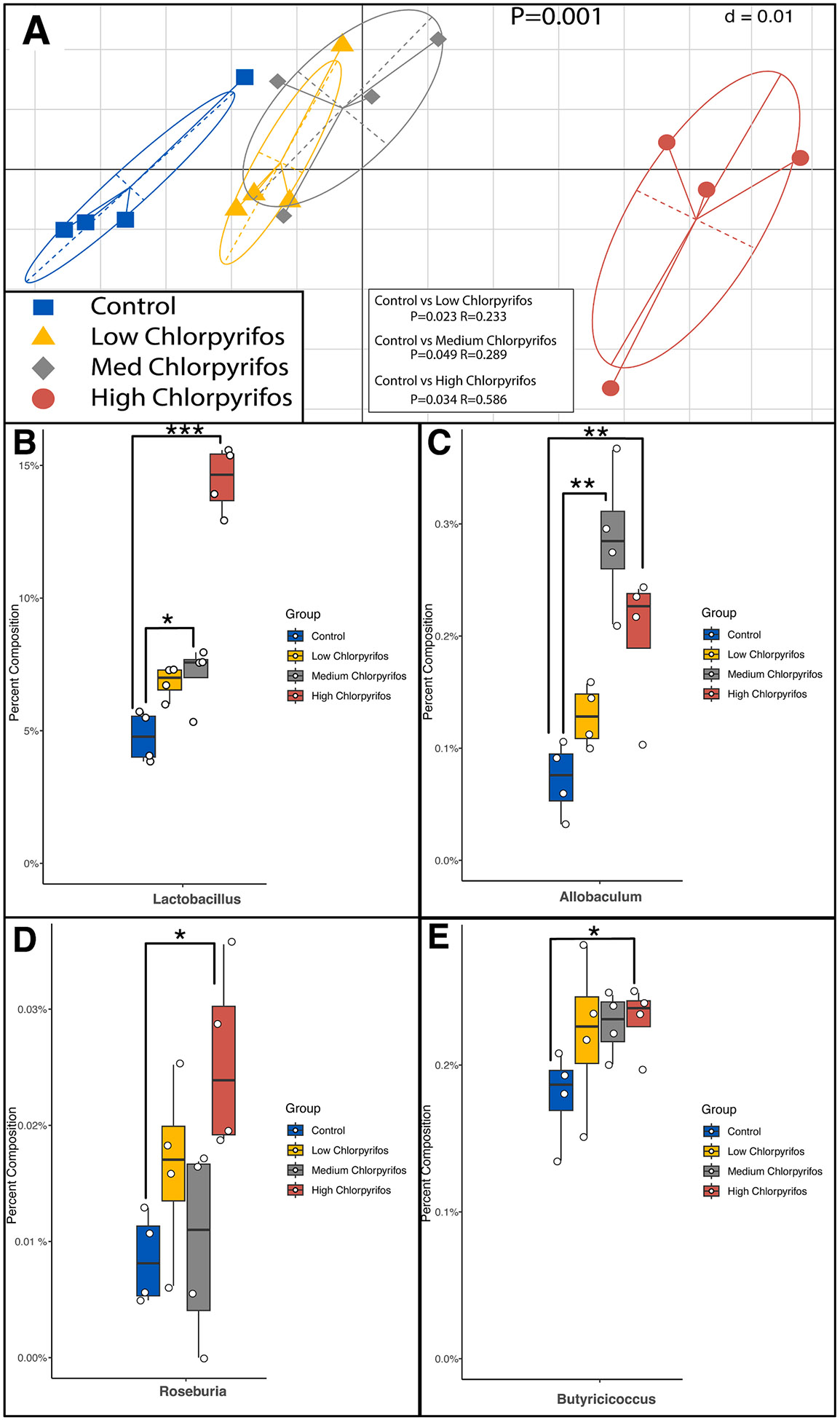
16S sequencing shows that chlorpyrifos causes significant shifts in the microbiome community structure. **A)** All chlorpyrifos doses result in significant bacterial community shifts as seen in the UniFrac distance plot which measures beta diversity (the GUnifrac R Package). Significance was obtained using permutational multivariate ANOVA (adonis2 package in R) with pairwise adonis to look at individual significance (pairwise.adonis package in R). The overall P value for the model was P=0.001. P<0.05 for all groups tested when compared to the control group. **B)** High and medium chlorpyrifos doses cause an increase in the relative abundance of the *Lactobacillus* genera. **C)** High and medium chlorpyrifos doses cause an increase in the relative abundance of the *Allobaculum* genus. **D)** The high chlorpyrifos dose causes an increase in the relative abundance of the *Roseburia* genus. **E)** The high chlorpyrifos dose causes an increase in the relative abundance of the *Butyricicoccus* genus. Significance for **B-E** was obtained ALDEx2 (Welch *t* test) analysis (P<0.1) and N=4 for all groups. Abundance results are illustrated as percent of the total relative abundance for each sample.

To identify whether chlorpyrifos exposure results in a shift in microbiome composition, we analyzed taxonomic differences between doses. MiSeq Nano 16S rRNA gene amplicon sequencing (16S) resulted in an average of 31,000 reads per sample with a standard deviation of 2,400 reads. Bacterial population shifts were measured with Unifrac distances using the GUniFrac package in R (Fig. 3A). Permutational multivariate analysis of variance using distance matrices (Adonis.3 in R) was used to find the overall p-value of the model (p = 0.001). To further investigate these relationships, Pairwise adonis (pairwise.adonis in R) was done using a false discovery rate used to adjust the p values which showed significance in low dose (0.01 mg/ml) chlorpyrifos, medium dose chlorpyrifos (0.1 mg/ml), and high dose chlorpyrifos (1 mg/ml) (p < 0.05 for all comparisons).

The 16S taxonomic analysis revealed shifts in the relative abundance of several bacterial genera. At the genus level, *Lactobacillus* and *Allobaculum* significantly increased (ALDEx2 (Fernandes et al., 2013) Welch’s T Test, p < 0.1,) after medium and high dose chlorpyrifos exposure compared to controls (Fig. 3B and C). *Roseburia* and *Butyricicoccus* both significantly increased (ALDEx2 (Fernandes et al., 2013) Welch’s T Test, p < 0.1) after high-dose chlorpyrifos exposure compared to controls (Fig. 3D and E). The significant changes in the abundance to a modest number of bacterial taxa between groups suggests that the metabolic differences observed via ^1^H NMR were not primarily caused by chlorpyrifos-induced alterations in microbial composition, but, more likely, by changes in metabolism within the population.

### 3.4. Gene marker prediction analysis with PICRUSt2 complements the metabolic results

**Fig. 4.**
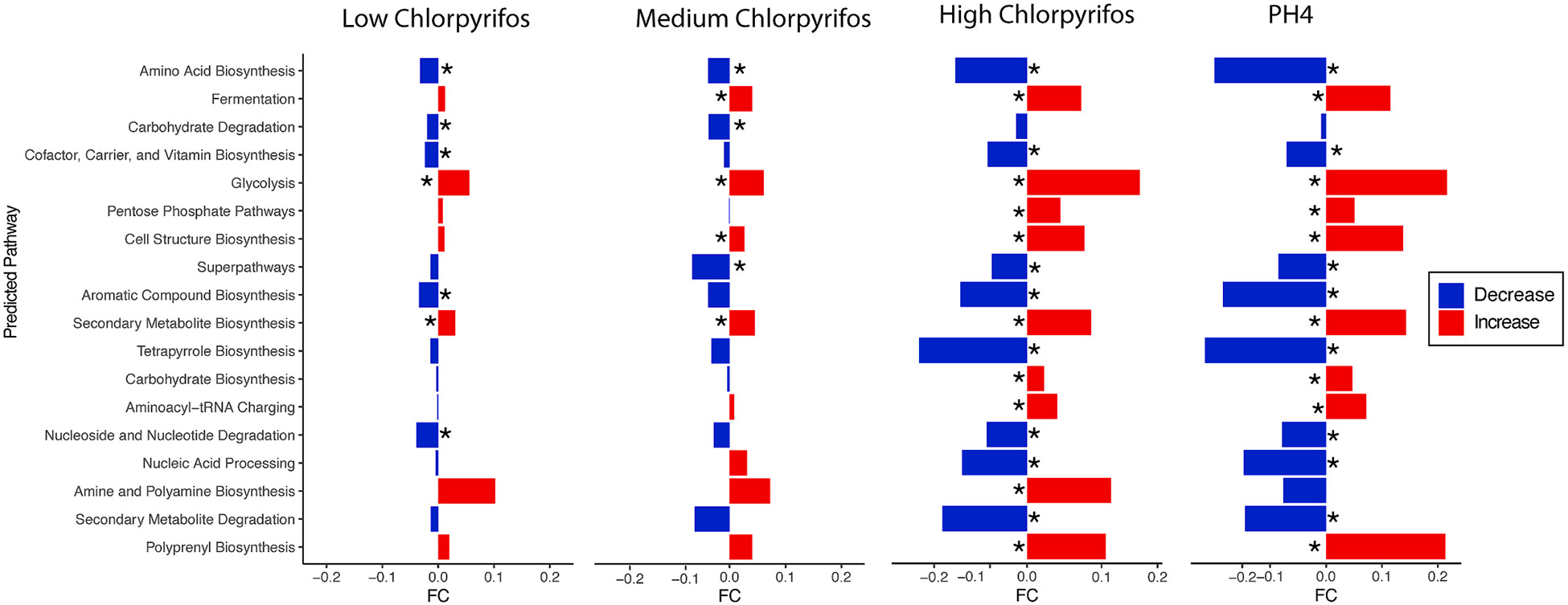
Significant bacterial functional changes caused by chlorpyrifos can be predicted with PICRUSt2 analysis. PICRUSt2 results can be combined into metacyc superpathways. Several, potentially dose-dependent, superpathways are predicted to significantly change after chlorpyrifos exposure. Significantly different metacyc superpathways are illustrated in terms of fold change difference. Fold change and significance were obtained from a DESeq2 analysis with Bonferroni p-value correction. *, p < 0.05. All red bars indicate a decrease when compared to control and all blue bars indicate an increase when compared to control.

To identify whether the shifts in metabolites observed in ^1^H NMR-based metabolomic analysis were consistent with the shifts in composition between groups, we predicted functional potential within each group using PICRUSt2 (Douglas et al., 2020). Pathway data from PICRUSt2 was condensed into MetaCyc superpathways. Relative abundance of bacterial genes that encode for amino acid biosynthesis were significantly predicted to decrease across all chlorpyrifos exposed groups and pH4 when compared to the control group (DESeq2 and Bonferroni correction, p < 0.05 for all groups) (Fig. 4). Relative abundance of bacterial genes that encode for cofactor, carrier and vitamin biosynthesis, and aromatic compound biosynthesis were significantly predicted to decrease in the low-dose (0.01 mg/ml) chlorpyrifos, high-dose chlorpyrifos (1 mg/ml), and the pH4 groups as compared to the controls (DESeq2 and Bonferroni correction, p < 0.05 for all groups). Conversely, relative abundance of bacterial genes that encode for glycolysis were predicted to significantly increase across all chlorpyrifos-exposed groups and the pH4 group when compared to the control group (DESeq2 and Bonferroni correction, p < 0.05 for all groups). Relative abundance of bacterial genes that encode for fermentation were also predicted to be increased in the chlorpyrifos groups, but this was only significantly predicted to increase in the medium and high chlorpyrifos groups and the pH4 group as compared to the control group (DESeq2 and Bonferroni correction, p < 0.05 for all groups). Relative abundance of bacterial genes that encode for carbohydrate biosynthesis were also significantly predicted to increase in the high-dose chlorpyrifos group and pH4 group when compared to the control group (DESeq2 and Bonferroni correction, p < 0.05 for all groups). Overall PICRUSt2 predicted major decreases in the relative abundance of bacterial genes that encode for several biosynthesis pathways (like amino acids, cofactors and aromatic compounds) and predicted increases of glycolysis and fermentation after chlorpyrifos exposure, again pointing to an overall decrease in bacterial metabolism.

**Fig. 5.**
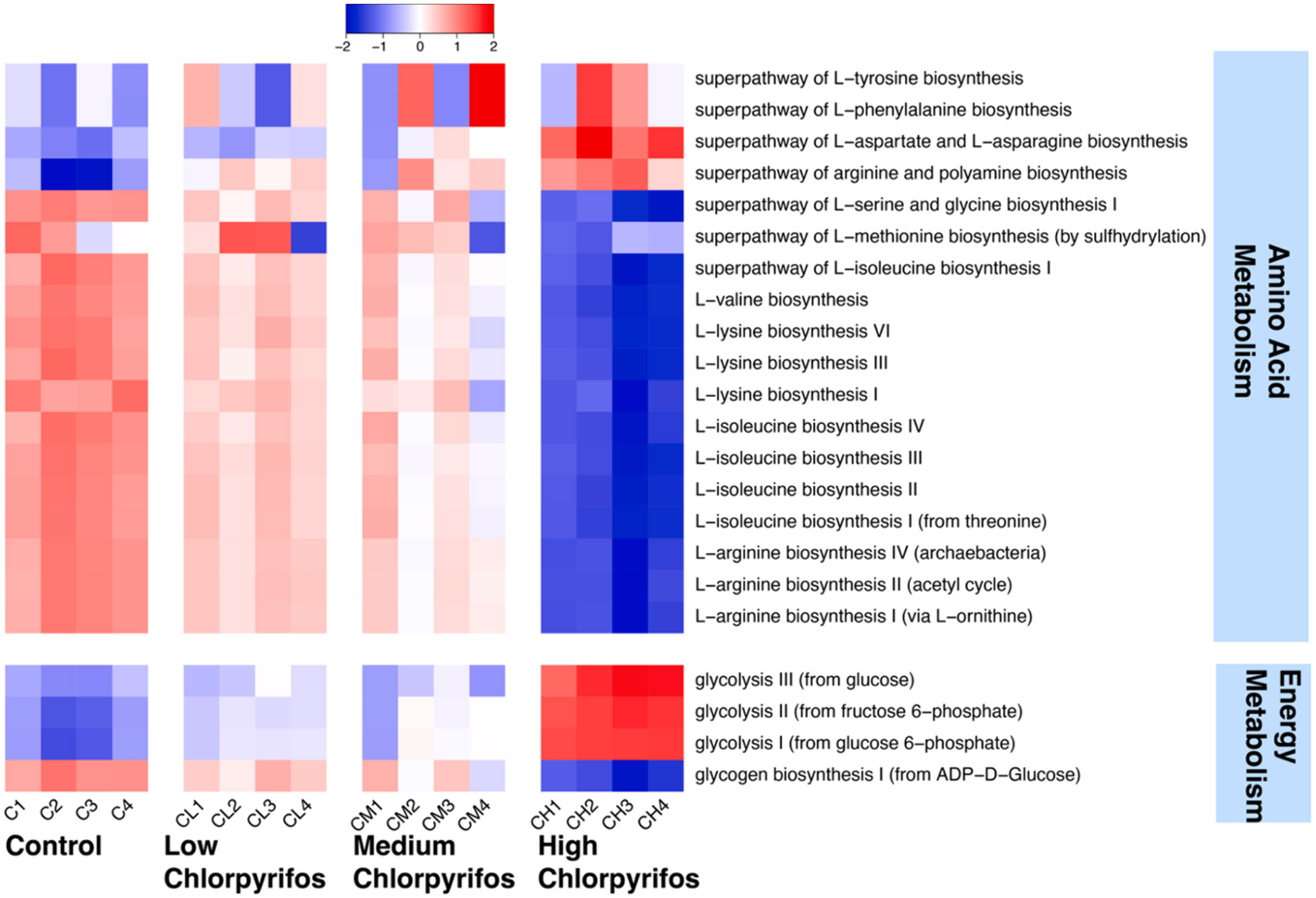
Individual predicted PICRUSt pathways can be used to confirm amino acid metabolomic results. This figure shows a heatmap of significantly different PICRUSt pathways which match the reported metabolomic changes from Fig. 2. Values are represented as Z scores with the equation $Z=\frac{x-\mu }{\sigma }$. Where x is the observed value, μ is the mean of the sample, and σ is the standard deviation of the sample. Red colors represent an increase compared to the average metabolite level and blue colors represent a decrease compared to the average metabolite level. Significance was obtained with a DESeq2 analysis with Bonferroni p-value correction. *, p < 0.05.

Individual pathways of note were examined and linked with the metabolomic data (Fig. 2). All the amino acids listed in Fig. 2 have predicted modulations in their respective biosynthesis pathways after PICRUSt2. Fig. 5 shows matching predicted amino acid biosynthesis pathway shifts for previously obtained metabolomic data. The predictions of the relative abundance of bacterial genes that encode for serine and glycine biosynthesis I, L-lysine biosynthesis III, L-methionine biosynthesis, L-arginine biosynthesis I, L-valine biosynthesis, and L-isoleucine biosynthesis II are significantly predicted to decrease in the high chlorpyrifos group as compared to controls (DESeq2 and Bonferroni correction, p < 0.05 for all groups) (Fig. 5). Additionally, relative abundance of bacterial genes that encode for L-arginine biosynthesis I, L-valine biosynthesis, and L-isoleucine biosynthesis II are predicted to significantly decrease in the medium-dose chlorpyrifos group when compared to control (DESeq2 and Bonferroni correction, p < 0.05 for all groups) (Fig. 5).

Relative abundance of bacterial genes that encode for glucose metabolism were predicted to decrease after high-dose chlorpyrifos and several glycolysis and glycogen biosynthesis pathways were predicted to change after PICRUSt2 analysis and are reported in Fig. 5. Relative abundance of bacterial genes that encode for glycogen biosynthesis I were predicted to significantly decrease in the high-dose chlorpyrifos group (DESeq2 and Bonferroni correction, p < 0.05 for all groups) (Fig. 5). There was a predicted significant dose-dependent increase in the relative abundance of bacterial genes that encode for Glycolysis I after chlorpyrifos exposure (DESeq2 and Bonferroni correction, p < 0.05 for all groups) (Fig. 5). Relative abundance of bacterial genes that encode for glycolysis III were predicted to significantly increase in the high-dose chlorpyrifos group (DESeq2 and Bonferroni correction, p < 0.05 for all groups) (Fig. 5).

## 4. Discussion

We investigated the direct effects of chlorpyrifos on the gut microbiota at doses nearing the EPA acute RfD of 5 μg/kg/day including low (**0.01 mg/mL**), medium (**0.1 mg/ mL**), and high (**1 mg/mL**) dose exposure. All three doses of chlorpyrifos caused significant changes to the gut microbiota at the bacterial community and metabolic levels.

Flow cytometric-based analyses of the gut microbiota have been used previously to characterize the direct effects of environmental chemicals on the gut microbiota (Maurice and Turnbaugh, 2013; Cai et al., 2018). Our study demonstrates that high doses of chlorpyrifos resulted in a significant decrease of SYBR green stain, a marker of nucleic acid presence, regardless of membrane status. A decrease in the levels of SYBR green stain illustrates an overall decrease of nucleic acids present in these bacterial cells when exposed to high doses of chlorpyrifos, potentially due to nucleic acid degradation. DNA damage and DNA instability of host cells have been reported in studies examining similar doses of chlorpyrifos (Kopjar et al., 2018), but our results are the first to suggest that this could occur in the gut microbiota. To confirm DNA damage or instability future studies assessing genotoxicity of bacteria following chlorpyrifos are warranted like a **T**erminal deoxynucleotidyl transferase (TdT) d**U**TP **N**ick-**E**nd **L**abeling (TUNEL) assay (Kyrylkova et al., 2012). Chlorpyrifos also appears to be bacteriostatic rather than bactericidal. This is evident by the non-significant changes with the propidium iodide and DiBAC stains and the dose-dependent decrease in the CFDA stain. CFDA enters the bacteria inert and requires bacterial metabolism to activate the dye and cause fluorescence. A decrease in CFDA stain equates to an overall decrease in cell metabolism caused by chlorpyrifos. Additionally, there was a decrease in the high nucleic acid content to low nucleic acid content ratio after chlorpyrifos exposure, which indicates cells are undergoing less replication and less cell metabolism (Gasol et al., 1999; Zubkov et al., 2001; Bouvier et al., 2007). Based on these observations, low doses of chlorpyrifos, close to the EPA’s allowable limit, have a direct effect on the gut microbiota.

These direct effects were further explored by measuring changes in the microbiota community with 16S rRNA gene amplicon analysis. Significant shifts to four bacterial taxa were caused by exposure to chlorpyrifos. Genera-level changes were seen in the medium and high doses of chlorpyrifos. Relative abundance of *Lactobacillus* increases nearly three-fold after exposure to chlorpyrifos. Enrichment and depletion of *Lactobacillus* have been reported in several diseases; *Lactobacillus* was seen to increase in Crohn’s disease (Lewis et al., 2017) and rheumatoid arthritis (Zhang et al., 2015), and *Lactobacillus* was seen to decrease in diseases like type two diabetes (Forslund et al., 2016) and colon cancer (Borges-Canha et al., 2015). From these previous studies, Lactobacillus has an important role in gut health (Lewis et al., 2017; Zhang et al., 2015; Forslund et al., 2016; Borges-Canha et al., 2015). Since chlorpyrifos has an effect on *Lactobacillus*, it is possible that chlorpyrifos exposure could be related to the onset of some of these diseases, but more work is needed to investigate this connection. *Allobaculum* also increased in relative abundance after chlorpyrifos exposure. Many *Allobaculum* species contain mucus-degrading enzymes which have been related to the onset of inflammatory bowel disease (van Muijlwijk et al., 2021). Relative abundance of short-chain fatty acid (SCFA) producing bacteria like *Roseburia* and *Butyricicoccus* were also found to increase after exposure to high dose chlorpyrifos. *Roseburia* is a major producer of propionate and butyrate (Koh et al., 2016) and *Butyricicoccus* is a major producer of butyrate (Boesmans et al., 2018). Conversely to the potential negative effects seen from increases of *Lactobacillus* and *Allobaculum*, the SCFA-producing bacteria are generally thought to have an anti-inflammatory effect and promote gut health (Martin-Gallausiaux et al., 2021). Even after a short incubation time (four hours), there were significant shifts of several genera after exposure to chlorpyrifos. This collectively suggests that even low doses of chlorpyrifos can have a significant effect on the gut microbiota community.

Metabolomics and the predictive software PICRUSt were used to investigate shifts in the bacterial metabolic profile caused by chlorpyrifos exposure. Significant increases in amino acids were noted by metabolomic analysis but PICRUSt predicted significant lower relative abundances of the genes that encode for the respective amino acid biosynthetic pathways. While this may seem counterintuitive, we propose that chlorpyrifos may prevent the gut bacteria from utilizing the amino acids for downstream metabolism, which matches the observation of the significant decrease in overall cell metabolism seen with the flow cytometry results. This would result in a surplus of amino acids (observed in this study) while also causing a decrease in the respective biosynthesis pathways (predicted in this study). This notion is further supported by the predicted decreases of other biosynthetic pathways like cofactor, carrier and vitamin biosynthesis, aromatic compound biosynthesis, and tetrapyrrole biosynthesis after chlorpyrifos exposure.

Along with a decrease in amino acid biosynthesis, chlorpyrifos may also affect bacterial glucose utilization. The relative abundance of the genes that encode bacterial glycolysis were predicted to significantly increase, and the relative abundance of glycogen biosynthesis were predicted to significantly decrease after chlorpyrifos exposure. These predicted results can be combined with the metabolomic results which show significant increases in glucose and succinate after chlorpyrifos exposure. Potentially the bacterial cells do not have a surplus of glucose to store and instead need to use what is available. This could also mean that bacteria are absorbing more glucose from the diet and less glucose is escaping to be absorbed by the host. It has also been reported that chlorpyrifos (5 mg/kg) can promote obesity and insulin resistance by increasing gut inflammation in mice (Liang et al., 2019). The relationship between chlorpyrifos, bacterial metabolism, and bacterial glucose levels could have a very important effect on host glucose levels and insulin sensitivity which needs to be further explored in subsequent studies.

There are, however, several important limitations of our study. First, since this is an *in vitro* system there is no host, so the current study misses potential host contributions that could impact the gut microbiota (e.g., changes in oxidative stress). Additionally, the *in vitro* system is not a perfect replication of the gut microbiome and will not sustain the species of the gut microbiome over long periods of time, this limits our exposure time to four hours. Lastly the doses used, while an approximation of the EPA acute reference dose, are likely higher in our system than they would be in the gastrointestinal tract of a mouse model. Future studies are needed to address these important questions.

Chlorpyrifos was banned in December 2022 but that decision was reversed in November of 2023 (Red River Valley Sugarbeet Growers, 2023). This study highlights the importance of studying pesticide doses at or close to the EPA reference dose and the importance of examining the effects of pesticides on the gut microbiota. The Human Health Benchmarks for Pesticides are EPA benchmarks of pesticide levels in drinking water that are the minimum dose that results in adverse effects. These benchmarks have been updated every four years (updated in 2013, 2017, and 2021) but the microbiota has not been taken into account when these benchmarks are created or updated. In fact, the cited pesticide risk assessment protocol for creating these benchmarks was created in October 1999, which is well before much of the research on the gut microbiome was completed (EPA’s Risk Assessment Process For Tolerance Reassessment, 1999). Unfortunately the current protocols for risk assessment for pesticide exposure do not take into account the gut microbiome. This study illustrates the importance of including the gut microbiota for future pesticide risk assessment.

## Supplementary Material

fx1

fx2

## Data Availability

Data will be made available on request.
