# Supplementary figures and images for: Chlorpyrifos modulates the mouse gut microbiota and metabolic activity
Source: Environ Int. Author manuscript; Available in PMC 2024 Nov 22. (PMC11583244; doi:10.1016/j.envint.2024.109022)

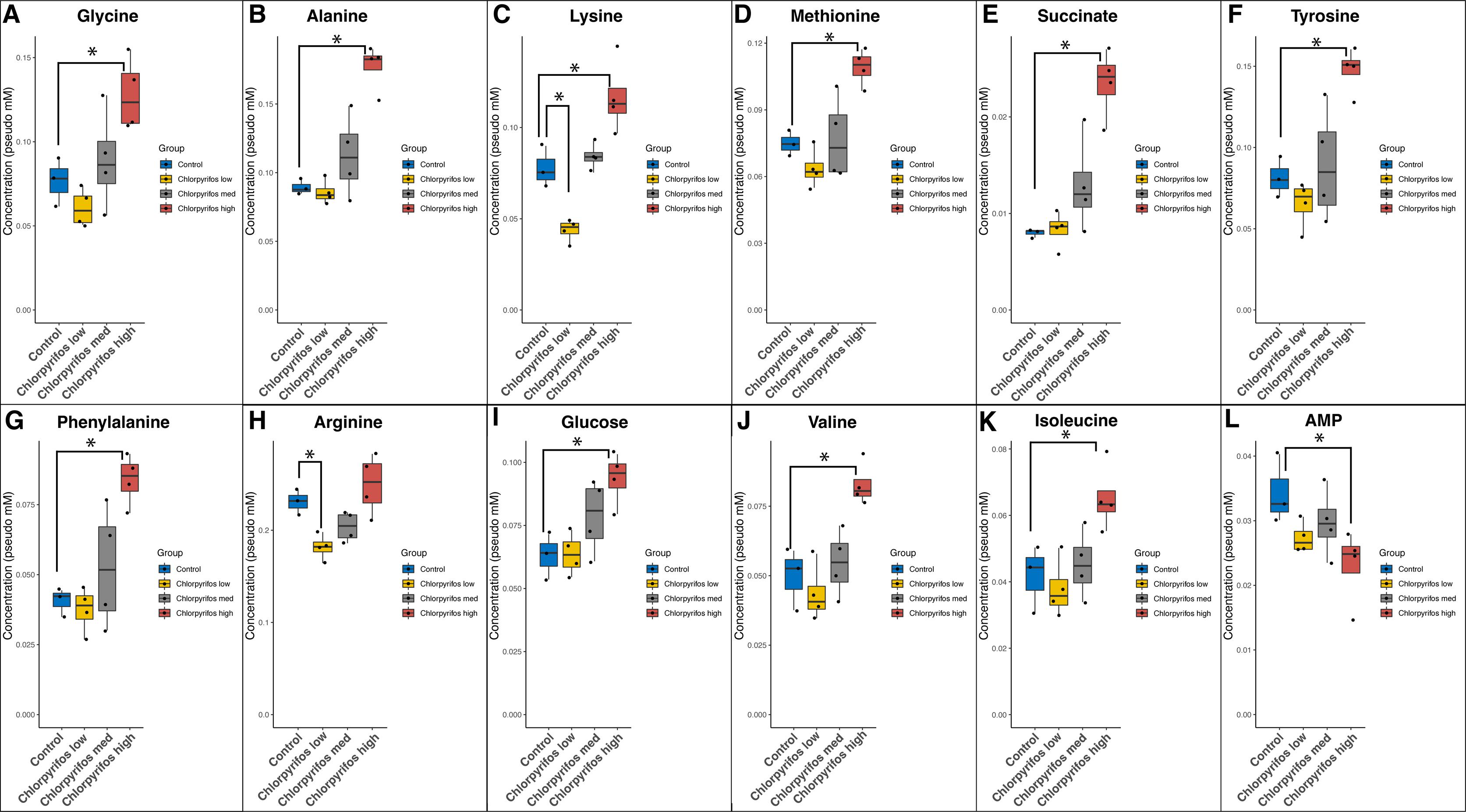

Supplement: fx1 [file NIHMS2031339-supplement-fx1.jpg]

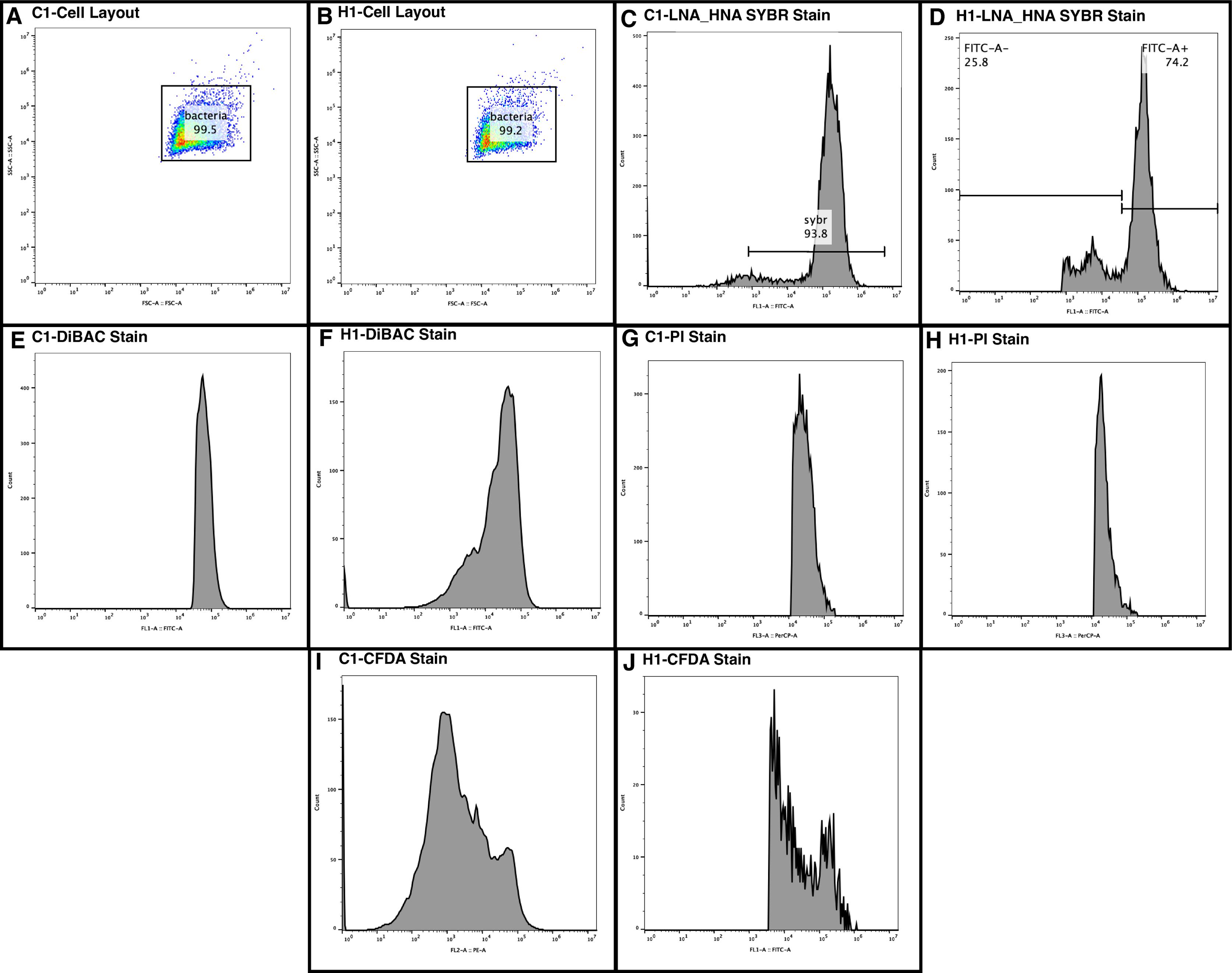

Supplement: fx2 [file NIHMS2031339-supplement-fx2.jpg]
